# Impaired Early Attentional Processes in Parkinson’s Disease: A High-Resolution Event-Related Potentials Study

**DOI:** 10.1371/journal.pone.0131654

**Published:** 2015-07-02

**Authors:** Perrine Bocquillon, Jean-Louis Bourriez, Ernesto Palmero-Soler, Luc Defebvre, Philippe Derambure, Kathy Dujardin

**Affiliations:** 1 Clinical Neurophysiology Department, Lille University Medical Center, Lille Cedex, France; 2 Inserm, U1171, Troubles cognitifs dégénératifs et vasculaires, Université de Lille, Lille Cedex, France; 3 Eemagine Medical Imaging Solutions GmbH, Berlin, Germany; 4 Neurology and Movement Disorders Department, Lille University Medical Center, Lille Cedex, France; University of Verona, ITALY

## Abstract

**Introduction:**

The selection of task-relevant information requires both the focalization of attention on the task and resistance to interference from irrelevant stimuli. A previous study using the P3 component of the event-related potentials suggested that a reduced ability to resist interference could be responsible for attention disorders at early stages of Parkinson’s disease (PD), with a possible role of the dorsolateral prefrontal cortex (DLPFC).

**Methods:**

Our objective was to better determine the origin of this impairment, by studying an earlier ERP component, the N2, and its subcomponents, as they reflect early inhibition processes and as they are known to have sources in the anterior cingulate cortex (ACC), which is involved together with the DLPFC in inhibition processes. Fifteen early-stage PD patients and 15 healthy controls (HCs) performed a three-stimulus visual oddball paradigm, consisting in detecting target inputs amongst standard stimuli, while resisting interference from distracter ones. A 128-channel electroencephalogram was recorded during this task and the generators of the N2 subcomponents were identified using standardized weighted low-resolution electromagnetic tomography (swLORETA).

**Results:**

PD patients displayed fewer N2 generators than HCs in both the DLPFC and the ACC, for all types of stimuli. In contrast to controls, PD patients did not show any differences between their generators for different N2 subcomponents.

**Conclusion:**

Our data suggest that impaired inhibition in PD results from dysfunction of the DLPFC and the ACC during the early stages of attentional processes.

## Introduction

Attention underlies most cognitive processes and can be focused by relevant signals derived from task demands (i.e. target stimuli) or captured by salient properties of stimuli that are sometimes irrelevant for the task (i.e. distracter stimuli) [1]. Input selection (as described by Luck and Gold [2]) enables the preferential processing of some information sources at the expense of others. According to Luck and Gold, this process can be subdivided into the control of selection (i.e. the process of determining which inputs should be selected) and the implementation of selection. The latter corresponds to the process of enhancing the target inputs while suppressing the distracter inputs. An impaired implementation of selection could then result from either a lack of target enhancing or a weaker ability to resist interference from distracter stimuli, or a mixed dysfunction of both mechanisms. It prevents from correctly performing a task in which distracter and target stimuli are intermixed.

Impairments in both of these mechanisms have been observed in Parkinson’s disease (PD) [3–6], a neurodegenerative disease characterized by loss of dopaminergic cells in the substantia nigra pars compacta. Parkinson’s disease can cause both motor symptoms, variously including bradykinesia, rest tremor and rigidity [7] and non-motor symptoms, including attention disorders—even in the early stages of the disease [8, 9]. These attention disorders are often associated with a dysexecutive syndrome [10]. Namely, PD patients were reported to present with an exaggerated bottom–up and/or attenuated top–down attentional control [5–6] that could contribute to their attention disorders.

Cognitive event-related potentials (ERPs) are frequently used to study attention processes, particularly during three-stimulus oddball paradigms in which a subject has to detect low-probability, awaited stimuli (i.e. targets) mixed randomly with low-probability, unexpected non-targets (i.e. distracters) and high-probability, expected non-targets (i.e. standard stimuli) [11–13]. Apart from the latency and amplitude data, the application of distributed source localization methods, such as standardized-weighted low resolution electromagnetic tomography (swLORETA) [14, 15] to ERPs has provided a critical tool for investigating the involvement of cortical networks in attentional processes. A previous study of P3 generators modifications in PD [16] during a three-stimulus oddball paradigm has suggested that a dysfunction of the dorsolateral prefrontal cortex (DLPFC) at late attention stages could be responsible for the lack of inhibition of irrelevant stimuli in PD. Indeed, despite few inter-group differences in P3 latency or amplitude, PD patients displayed fewer P3 generators in the DLPFC but after distracter presentation only. This finding suggested that lesser recruitment of the DLPFC may impair the inhibition of irrelevant stimuli and could thus be responsible for the attention impairments observed in PD, in agreement with previous studies [5–6]. However, the exact cause of this DLPFC dysfunction remains unknown; it could be directly related to an abnormal connection with the associative striatum or may depend on connections between the associative striatum, the DLPFC and other areas. Given that we evidenced a specific impairment in distracter inhibition, one can suspect involvement of the anterior cingulate cortex (ACC). In fact, the ACC is a key structure in cognitive control and inhibition [17]. Moreover, it is known to interact with the DLPFC [18, 19]. In particular, event-related functional magnetic resonance imaging (fMRI) during a Stroop task has shown co-activation of the ACC and the DLPFC—suggesting that a conflict signal from the ACC may help to recruit additional cognitive control functions performed by the DLPFC [18, 20]. It has thus been supposed that the ACC has a role in inhibition, albeit during an early time window. Indeed, the ACC may be related to generation of the N2 [21]—the second characteristic negative peak seen in the cognitive ERP. The N2 usually occurs between 200 and 350 ms after a stimulus [21] and has been linked to inhibition processes [17]. If the ACC is indeed involved in inhibition, it should therefore act during this time window.

Two N2 subcomponents have been described in oddball paradigms: an anterior subcomponent (previously known as N2b) and a more posterior component (initially called N2c) [21]. The anterior N2 can be further divided into (i) a deviance-related N2 (also known as a “novelty N2”) that may correspond to detection of a mismatch between the presented and expected stimuli and (ii) a control-related N2 (also known as a “no-go N2”) that may predominantly reflect inhibition and conflict monitoring processes [17]. The posterior N2 has been less well characterized but is target-specific [21, 22] and could reflect classification of these stimuli [21]. N2 features are less known than P3 ones in PD. Some studies showed a longer latency [23–32] and a lower amplitude [23, 26, 33–35], relative to HCs. But these results are still controversial since other recent studies of the no-go N2 in the frontal areas highlighted greater amplitudes in these regions in PD patients [24, 36]. To the best of our knowledge, source analyses of N2 subcomponents have never previously been performed in PD patients performing an oddball paradigm.

The objective of the present study was thus to better determine the origin of the attentional impairment in early PD, as shown in our previous study of P3 components [16], by combining an investigation of the N2 components' usual features (amplitude and latency) with identification of their cortical generators in a swLORETA source analysis [15, 37]. If the impairment in inhibition specifically concerns the implementation of selection at the P3 time-window, with a decision to suppress distracter inputs from the DLPFC, one would not expect to see a difference between HCs and PD patients in terms of the N2 characteristics. However, if it is due to an earlier impairment in cognitive control and in mismatch detection, intergroup differences should be seen for all N2 subcomponents; in particularly, PD patients and HCs should differ in terms of N2 generators in frontal areas (including the ACC).

## Material and Methods

### Participants

Our study included 15 right-handed patients (ten males and five females) with probable PD (diagnosed according to international criteria [38]). All patients were assessed after administration of their usual anti-parkinsonian medication (eight were taking dopaminergic agonists only, two were on L-dopa only and five were on a combination of dopaminergic agonists and L-dopa). The mean L-dopa equivalent daily dosage is shown in Table 1 [39]. We excluded patients with motor fluctuations or a tremor subscore (items 20 and 21) greater than 2 on the UPDRS III scale, those receiving deep brain stimulation or those suffering from depression or dementia (according to the DSM IV-TR [40] and PD dementia criteria [41], respectively). Fifteen right-handed, HCs (eight males and seven females) also participated in the study. According to self-reports, the subjects had no history of psychiatric problems and were not taking any psycho-active drugs. The HCs were also free of neurological disease. The two groups were matched in terms of age, gender and duration of formal education. Table 1 summarizes the subjects’ demographic and clinical features.

**Table 1:** Clinical and demographic features of the Parkinson's disease (PD) patients and healthy controls.

	PD patients	Healthy controls	p
Age (years)	59.2 (6.4)	59.1 (7.4)	0.979
Gender ratio (M/F)	10/5	8/7	0.456
Duration of education (years)	12.5 (2.4)	12.7 (3.2)	0.966
Mattis Dementia Rating Scale (out of 144)	141.3 (2.7)	142.1 (1.6)	0.642
Montgomery-Asberg Depression Rating Scale score	3.1 (2.2)	2.2 (4.5)	0.029
Hoehn and Yahr score	1.5 (0.5)		
UPDRS III score	18.6 (8.7)		
Mean (SD) L-dopa equivalent daily dose (mg/d)	542 (222)		
Time since disease onset (years)	4.8 (3.5)		

Mean (standard deviation). p values were determined in t-tests (except for the gender ratio, to which a χ^2^ test was applied).

All participants were free of visual impairments, according to the Early Treatment Diabetic Retinopathy Study scale [42]. An extensive cognitive examination (including an assessment of the overall cognitive status [43] and the main cognitive domains (see S1 File for procedure and results and S1 Table) enabled us to rule out cognitive decline or dementia. The severity of anxious-depressive symptoms was assessed on the Montgomery-Asberg Depression Rating Scale [44].

### Description of Procedures

#### Task and recording procedure

**Fig 1:**
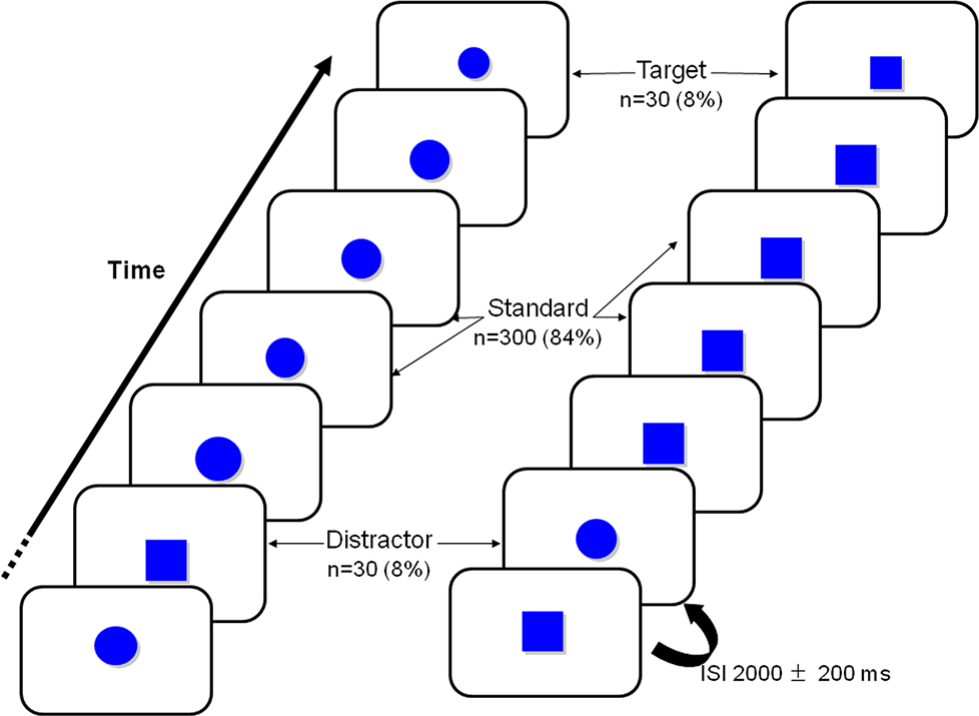
A schematic representation of the three-stimulus visual oddball paradigm. The circle task is on the left and the square task on the right.

Subjects were comfortably seated and watched a 17-inch computer monitor set 150 cm in front of them at head height. Event-related potentials were recorded as the subjects performed a three-stimulus visual oddball task similar to that used by Bledowski et al. [45]. A session included two different task types (a circle task with squares as distracters and a square task with circles as distracters) with 360 stimuli each. The order of the two task types was counterbalanced so that half the participants saw circles first and half saw squares first. Fig 1 depicts the experimental task: the stimuli were solid blue shapes displayed in a semi-random order for 75 ms each. The interstimulus interval varied from 1800 to 2200 ms. The stimuli were defined as standard shapes (40 mm diameter circles or 35 mm sided squares), distracters (a different shape: 35 mm sided squares or 40 mm diameter circles, respectively) or targets (smaller than the standard shape: 33 mm diameter circles or 30 mm sided squares) and were displayed with a probability of 0.84, 0.08, and 0.08, respectively. The subject was told to respond to presentation of a target stimulus by pressing a button with his/her right hand within 2000 ms. Before each task, all subjects had a practice run in the absence of distracter stimuli. The reaction time, omission rate and standard and distracter commission rates were recorded. The omission rate was defined as the number of misses divided by the total number of targets (i.e. 60) x 100. The overall commission rate was defined as the number of false alarms divided by the total number of non-target stimuli (distracter + standard stimuli, i.e. 660) x 100. The distracter commission rate corresponded to the number of false alarms after occurrence of a distracter divided by the total numbers of distracters (i.e. 60) x 100.

Electroencephalograms (EEGs) were recorded from 128 scalp sites, using a DC amplifier (ANT Software BV, Enschede, the Netherlands) and a Quick-cap 128 AgCl electrode cap (ANT Software BV) placed according to the 10/05 international system and with a linked mastoid reference [46]. The impedance was kept below 5 kΩ. An electro-oculogram (EOG) was recorded to detect artefacts related to eye movements and blinking. The EEG and EOG datasets were digitized with a sampling rate of 512 Hz and recorded with EEProbe software (ANT Software BV).

#### EEG analysis

The EEG datasets were analyzed with EEProbe software. The procedure was the same as that described in Bocquillon et al. [14]. The raw data waveforms were band-pass filtered by convolving them with a finite-impulse response filter and a Hamming window. The half-power cut-offs were 0.1 and 30 Hz. EEG epochs that contained eye movements or blink artifacts were automatically detected, then manually classified as either blinks or eye movements and separately corrected with the EEProbe regression algorithm. Whenever the subject missed a target stimulus or responded to a distracter stimulus, the event was excluded from the EEG analysis. The waveforms (analyzed from 100 ms pre-stimulus to 900 ms post-stimulus) were averaged separately for the standard, distracter and target conditions. For each epoch, a baseline correction was performed by using data from 100 ms prior to the stimulus. The N200 peak was defined as the largest negative deflection in the standard, distracter and target stimuli waveforms within the 160–400 ms time window and was thus referred to as the standard-, distracter- and target-elicited N200, respectively. The N200 amplitude was defined as the voltage difference between the baseline and the largest negative peak in the analyzed time window. Latency was defined as the time between stimulus onset and the largest negative peak. Amplitude and latency measures were performed for the three midline electrodes (Fz, Cz and Pz).

#### swLORETA N2 source localization

Source localization for N2 was performed according to the swLORETA procedure described in our previous study of P3 [14].

The swLORETA solutions were computed with ASA software (ANT Software BV) for each time point within a 40 ms time window around the N2 peak (referred to as the “peak window”) in each condition and each participant. We then calculated the mean value of the swLORETA analysis for all time points. The same calculation was performed within a 40 ms time window during the baseline period (-70 to -30 ms, referred to as the “baseline window”). The swLORETA solutions were computed using a three-dimensional grid of points (or voxels) representing the signal’s possible sources. Furthermore, solutions were restricted to the grey matter by selecting only voxels in which the grey matter probability was not equal to zero (based on the probabilistic brain tissue maps available from the Montreal Neurological Institute [47–49]). Lastly, the 1056 grid points (with a 5 mm grid spacing) and the recording array (128 electrodes) were registered against the Collins 27 MRI map [48]. The boundary model was used to compute the lead field matrix and thus solve the inverse problem [50].

### Ethics

All study subjects provided their written, informed consent to participation and the study had been approved by the local institutional review board (“Comité de Protection des Personnes Nord-Ouest IV”, 2007-A 00227–46).

### Statistical Analysis

#### Behavioral data

Due to a floor effect and the skewness of the distributions, data were described by median and range values and Mann-Whitney tests were used to compare reaction times, omission rates and overall and distracter commission rates in PD patients and HCs. The significance threshold was set to p<0.05.

#### Amplitude and latency data

Two-factor, repeated-measures analyses of variance (ANOVAs) were performed, with the stimulus type (standard, distracter or target) and location (Fz, Cz and Pz) as within-group factors and the group as between-subjects factor. A Greenhouse-Geisser correction was applied when the assumption of sphericity was not met. When necessary, post-hoc analyses with paired-t tests were performed. The threshold for statistical significance was set to p<0.05.

#### Source localization data

One-tailed t-tests were performed for each subject for each voxel of the source space (i.e., 1056 t-tests in total). Given that (i) the central limit theorem cannot rule out an effect of non-normality and (ii) it is difficult to prove that the modulus of the swLORETA solution follows a normal distribution (especially in experiments where there are relatively few degrees of freedom), it is necessary to use a statistical method that does not rely on an assumption of normality. Moreover, since we were performing 1056 simultaneous t-tests, we needed to control for the false positives that may result from performing multiple tests. The non-parametric permutation method [51] provides just such a framework and has been implemented by several authors in functional neuroimaging studies [52–54] and swLORETA analyses [55]. In contrast to parametric approaches (in which the statistic must have a known, null distributional form), the permutation approach uses the data itself to generate the probability distribution for testing the null hypothesis (for a highly detailed procedure and rationale, see Cebolla et al. [55]).

To locate standard-, distracter- and target-elicited N2 generators, we created difference images for each condition by subtracting the modulus of the mean swLORETA solution in the baseline window from the modulus at the peak window. We then used this difference image to compute a T-image (with a T value per voxel) by performing a one-tailed, paired t-test for each voxel of the source space; in the null hypothesis, the distribution of the voxel values from the subjects' difference images has a zero mean. However, instead of assuming a normal distribution when assessing the statistical significance of the T score at each voxel, we used the permutation method [51]. The threshold for statistical significance was set to p<0.001.

To compare the N2 generators from the various conditions, we applied a distracter-standard contrast (to assess specific generators of deviance N2) and a target-standard contrast (to highlight specific generators of the “go” N2) in each group.

Two sample t-tests were used to compare intensity-normalized swLORETA maps in patients with those in controls. This was done with a PD patient-HC contrast (in order to identify N2 generators found in PD patients but not in HCs) and with a HC-PD patient contrast (in order to highlight N2 sources only displayed by HCs).

The significance threshold was set to p<0.05 for two-sample t-tests and for paired t-tests.

The final x, y and z coordinates used to label the corresponding brain areas were based on the Talairach atlas. The coordinates were obtained by using ASA software to place the corresponding Talairach markers in the Collins 27 brain.

Hence, it was possible to obtain the Talairach coordinates of every voxel inside the brain for later comparison with a Talairach atlas [56, 57]. The coordinates correspond to the voxels that have a local maximum for t-values. To define a voxel as a local maximum, its t-value was automatically compared with the t-value displayed by its 16 nearest neighbors. If any of the neighbors had a higher t-value than the voxel under examination, the latter was not considered as a local maximum.

## Results

### Behavioral Results

According to Mann-Whitney tests, the two groups did not differ significantly in terms of median reaction time (PD patients: 556 ms (range 435–757) compared to HCs (550 ms (range 439–841), p = 0.756), omission rate (PD: 10% (range 0–28) vs HCs: 6.4% (range 0–23), p = 0.574), or overall commission rate (PD: 6% (range 0–38.9) vs HCs: 1.8% (range 0.6–9.1), p = 0.329). Mann-Whitney tests revealed a significantly higher median distracter commission rate (Z = -2.374, p = 0.018) in PD patients (0.5% (range 0–16.6)) than in HCs (0.0% (range 0–1.77)).

### N2 Amplitude and Latency

Mean (SD) latencies and amplitudes in Fz, Cz and Pz are detailed in S2 Table. The ERP waveforms at Cz are shown in S1 Fig.

ANOVA performed on the amplitude data revealed a significant main effect of stimulus (F(2,56) = 3.61, p = 0.047), with a larger N200 in the target condition than in the standard (F(1,28) = 8.1, p = 0.008) and distracter (F(1,28) = 4.193, p = 0.05) conditions. A main effect of location (F(2,56) = 4.30, p = 0.038) was also found, with a smaller N200 at Pz than at Cz (F(1,28) = 7.086, p = 0.014), with no other significant differences. No main effects of group or interaction were found, apart from a trend towards a group x location interaction (F(2,56) = 3.732, p = 0.053), with a larger N200 in anterior locations than in posterior locations in HCs (contrasting with the lack of a location-related difference in PD patients).

An ANOVA performed on the latency data did not reveal any significant main effects or interactions.

### Localization of N2 Cortical Generators with swLORETA

The Talairach coordinates of the cortical areas involved and the corresponding t-scores can be found in S3–S5 Tables.

#### Identification of the generators of the standard-elicited N2 subcomponent

**Fig 2:**
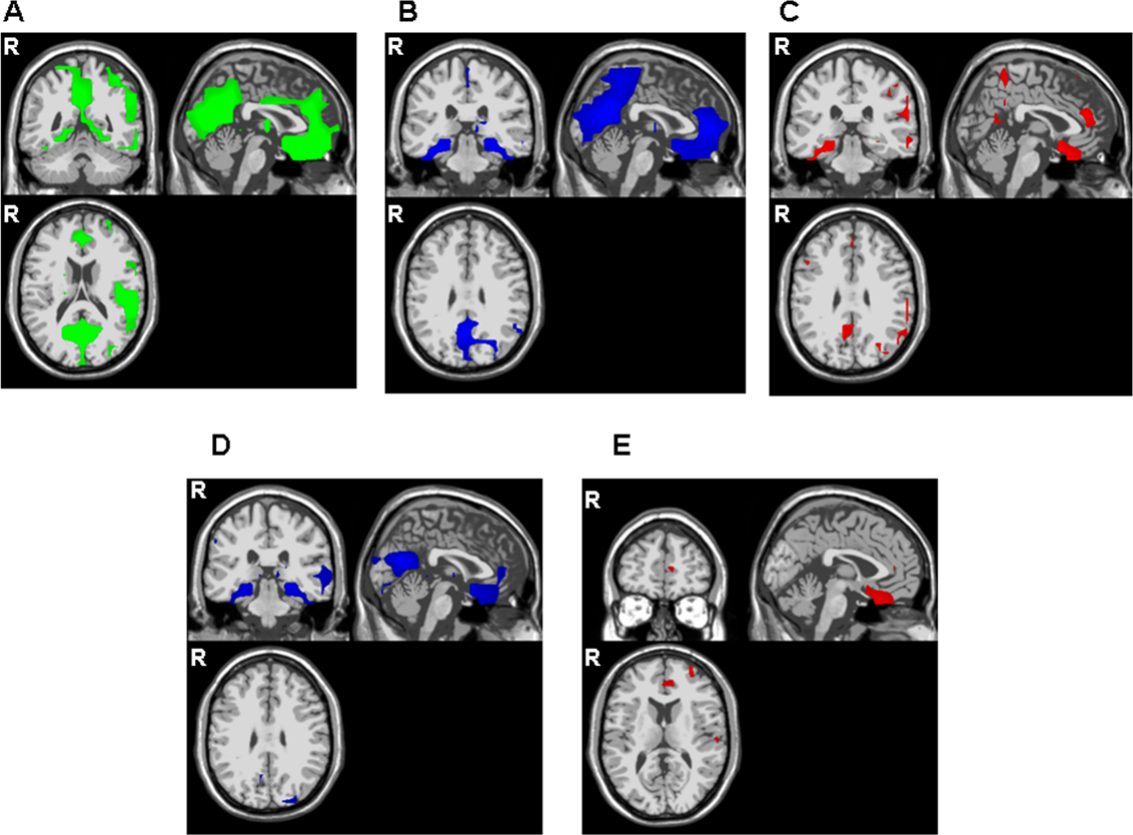
Statistical maps of the N200 components' grey matter current density obtained with swLORETA (healthy controls). Top panel (2A-C): identification of N2 generators for the standard-elicited, distracter-elicited and target-elicited N2 components, respectively (p<0.001). Bottom panel (2D-E): inter-condition comparisons of N2 generators in healthy controls. 2D: distracter-elicited vs. standard-elicited N200; 2E: target-elicited vs. standard-elicited N2 (p<0.05).

In HCs (Fig 2A), the standard-elicited N2 component was mostly generated within a bilateral, frontoparietal network—notably the frontal areas and the inferior regions in general, including the precentral area (BAs 4, 6, 9 and 10, BAs 7, 39 and 40). The ACC (BA 24) and the posterior cingulate cortex (PCC; BA 30) were also found to contain standard-N2 generators. A few sources were detected within the basal ganglia and the insula.

**Fig 3:**
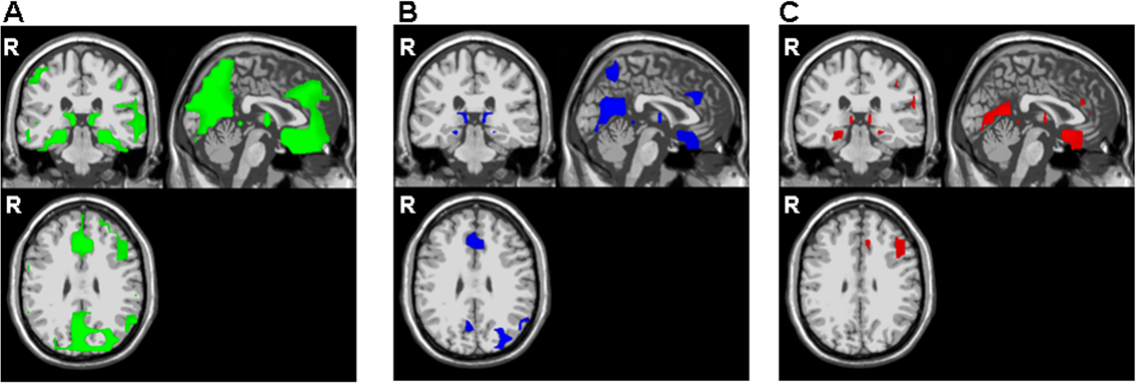
Statistical maps of the N2 components' grey matter current density obtained with swLORETA (Parkinson’s disease patients). Identification of N2 generators for standard-elicited (3A), distracter-elicited (3B) and target-elicited (3C) N2 components (p<0.001).

In PD patients (Fig 3A), the standard-elicited N2 component had sources in the ACC, the PCC, the insula and the basal ganglia. A few generators were also found in the frontal areas (BAs 6, 9, 45), inferior parietal areas (BAs 2, 40) and central precuneus (BA 7), although frontal sources were less predominant. Lastly, there were many standard-N2 generators in the occipital areas (BAs 18–19) and a number in the temporal lobes (BAs 20 and 22).

#### Identification of the generators of the distracter-elicited N2 subcomponent

In HCs, distracter-elicited N2 component generators were also found in the precentral gyri (BA 4), the left superior frontal gyrus (BA 6) and the anterior precuneus. Medial areas were also involved (including the PCC (BAs 30 and 31), the ACC and midcingulate (BA 32)), along with the basal ganglia (putamen, thalamus, caudate). As shown in Fig 2B, generators were also found in the occipital and temporal lobes and the right insula.

In PD patients, there were distracter-elicited N2 generators in the right middle frontal and prefrontal gyri (BA 6 and 8), the anterior precuneus (BA7), the PCC (BA 29), the ACC (BA 32) and the basal ganglia (the caudate and thalamus). Sources were also found in the occipital areas (BAs 18–19), the right middle temporal gyrus and the right insula (Fig 3B).

#### Identification of generators of the target-elicited N2 subcomponent

In HCs, the target-elicited N2 component was found to have sources in a large frontal network, including the precentral gyri (BAs 4 and 6), the bilateral superior (BAs 6, 8 and 10), right middle (BA 6) and left inferior (BA 9) frontal gyri and extending to the parietal lobe (the supramarginalis gyrus). Generators were also observed in the left ACC (BA 32) and medial frontal gyrus (BA 25), the bilateral temporal and occipital lobes and the right thalamus (Fig 2C).

In PD patients, generators were found in the right precentral and middle frontal gyri (BAs 6 and 9 respectively), the right postcentral gyrus (BA 2), the left inferior parietal gyrus (BA 40) and the bilateral superior temporal lobes (BAs 39 and 42) (Fig 3C). Sources were also observed in the ACC (BA 32), the occipital lobes (BA 18) and the basal ganglia (the putamen, caudate and thalamus).

#### Identification of “specific” distracter and target N2 generators

In HCs (Fig 2), the three N2 subcomponents had some common generators but distracter- or target-elicited N2 also had specific sources. Paired t-tests (comparing target- and distracter-N2 generators with standard-N2 generators) enabled us to identify areas that were more specific for distracter and target N2 generation, respectively. Fig 2D and 2E show the swLORETA t-test maps.

Specific distracter-elicited N2 generators were found in the left superior and right medial frontal lobe (mostly BAs 6 and 10), the precentral gyrus (BA 4), the right inferior parietal lobe (BA 40), the PCC (BA 30), the bilateral temporal lobes areas (BAs 20–22, 28, 36 and 39), the occipital lobes (BAs 18–19) and the right thalamus.

Specific target-elicited N2 generators were found in the left middle and right superior frontal gyri, the left precentral gyri (BA 6–10), the ACC (BA 32), the superior parietal lobe and supramarginalis gyrus (BA 7–40), the central precuneus (BA 19), the temporal lobe (BAs 21-28-36-37) and the occipital lobe (BAs 19–39).

In PD patients, there were no specific generators for distracter-, standard- or target-elicited N2s.

#### Comparison of N2 generators in HCs and PD patients

**Fig 4:**
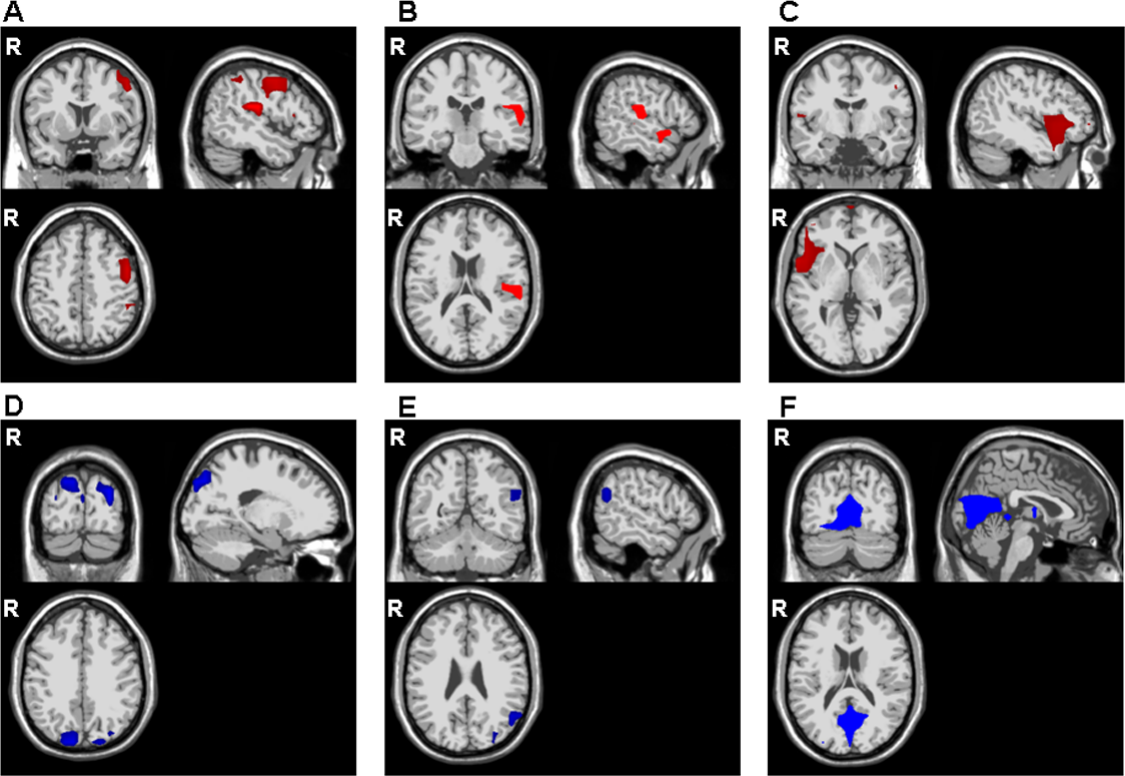
Between-group statistical maps of the N200 components' grey matter current densities, with the permutation method (p<0.05). Top panel (4A-C): healthy controls vs. PD patients (4A: standard N200, 4B: distracter N200, 4C: target N200). Bottom panel (4D-F): PD patients vs. healthy controls (4D: standard N2, 4E: distracter N2, 4F: target N2).

As shown in Fig 4A, application of a two-sample t-test with an HC-PD patient contrast to the standard-elicited N2 component revealed fewer generators in PD patients than in HCs in the precentral gyrus (BA6), the medial frontal areas (the left ACC (BA 32) and medial frontal lobe (BA 10)), the right inferior frontal gyrus (BAs 44 and 47), the right inferior parietal lobe (BA 40) and the left insula (BA 13). When applied to the distracter-elicited N2 (Fig 4B), the same contrast revealed differences in the frontal lobes (BAs 10 and 47), the ACC (BA 32) and the right superior temporal gyrus (BA 42). In HCs, target-elicited N2 generators (Fig 4C) were only observed in the left inferior and medial frontal gyri (BAs 10 and 45), the right precentral gyrus (BA 4) and the superior temporal gyri (BA 22).

When performing a two sample t-test with a PD-HC contrast (see Fig 4A), the standard-elicited N2 component showed more generators in PD patients in posterior areas, including the occipital lobes (BAs 18–19), the right central precuneus (BA 19), the left inferior parietal and postcentral lobes (BAs 1- and 40) and the left fusiforms gyrus (BA 37). The distracter-elicited N2 had more generators in PD patients in the right middle and superior occipital lobes (BA 19), the anterior precuneus (BA 7) and the right superior temporal lobe (BA 39) near the temporo-parieto-occipital junction (Fig 4E). As shown in Fig 4F, the target-elicited N2 generators that were more specific to PD patients were located in the occipital lobes (BA 18) and the left precentral and postcentral gyri (BAs 3–4).

## Discussion

The present study's primary objective was to use swLORETA to identify modifications of areas involved in the generation of N2 subcomponents during a three-stimuli oddball task in PD patients, in order to better elucidate the origin of the attention disorders in this disease. Our results showed that despite few inter-group differences in the classical features (i.e. amplitude and latency) of the N2 subcomponents, there were differences in the various generators' locations. Particularly, we found fewer N2 generators (for all subcomponents) in the PD patients' inferior and medial frontal lobes (BAs 10, 44–45 and 47), including the ACC (mostly BA 32)) and also (for standard and target-elicited N2 only) in the precentral gyrus (BAs 4 and 6). In contrast, PD patients had more N2 generators than HCs did in the occipital (BAs 18–19) and parietal lobes and in the temporoparieto-occipital junction (BAs 1, 37 and 39–40) for all N2 subcomponents and (for standard and distracter-elicited N2) the precuneus. Another important finding was the lack of difference between generators of the three N2 subcomponents in PD patients; this contrasted with the situation in HCs, who had distinct sources for specific distracter- and target-elicited N2s.

Even though behavioural results only show a mild deficit in inhibition of the distracter stimuli in PD patients, the swLORETA results suggest that in the initial stages of stimulus processing, PD patients could be impaired in mismatch detection and subsequent classification of the presented stimulus as standard, target and distracter. This constitutes more than just a dysfunction of response selection for target stimuli or of the decision to inhibit responses to distracter and standard stimuli. One can legitimately hypothesize that in early-stage processing, PD patients handle different types of stimuli in the same way. This is suggested by the fact that (i) in PD, there were no significant differences between the generators for the three conditions and (ii) HC vs. PD differences in the generators concerned the same areas of the brain in all three stimuli conditions. These abnormalities were nevertheless quite subtle, since HCs and PD patients did not differ significantly in terms of N2 latencies and amplitudes. However, we did see a trend towards a "location" x "group" interaction, with (i) larger N2s in frontocentral areas than in parietal areas in HCs only and (ii) the lack of a location effect in PD patients. The areas in which N2 generators were found in HCs but not in PD patients were primarily frontal. More precisely, these differences concerned the medial frontal cortex (including the ACC—mainly the BA 32 part) and the inferior frontal cortex. The medial frontal gyrus and the ACC have already been identified as key structures in N2 generation, in both dipolar and distributed source analysis studies [58–74]. Positron emission tomography (PET) and fMRI studies have also observed activation in these areas [62, 75–82]. The inferior frontal gyrus is also a putative N2 source, as already shown with dipolar [83] and distributed source analyses [67] and as suggested by event-related fMRI studies [75, 76, 78–81, 84–90] and PET studies [77]. These literature data strongly suggest that dysfunction of the inferior frontal cortex (i.e. part of the DLPFC) has a role in PD attention disorders, as already shown by the low number of distracter-elicited P3 generators in this area in PD patients [16]. Our present data further suggest that attentional impairment in PD concerns the early stages of attentional processing as well as the late stages, since we observed fewer N2 generators in the ACC in PD patients than in HCs. Hence, the ACC may be involved in disorders of early-stage attention processes in PD. Since the DLPFC and the ACC are both part of the corticostriatal loops involved in PD [91], the low number of N2 generators probably results from perturbation of these areas' connections with the associative striatum.

While PD patients displayed less N2 sources in these frontal areas than the HCs, N2 generators were unexpectedly found in posterior regions (mainly in the occipital lobe and at the temporoparieto-occipital junction). These posterior areas belong to the associative visual pathways (the secondary visual cortex and both the dorsal and ventral visual streams) and are usually involved in early visual processes [92, 93]. One explanation would be a delay in processing these visual stimuli. However, in such a case, the latencies of early components (like the N1 and P2) would have been affected; this was not observed. Another, more probable hypothesis relates to abnormal involvement of these areas, which are usually not involved in stimulus categorization.

This may reflect brain plasticity in response to frontal dysfunction in PD; in such a case, the observed N2 would have come from additional generators recruited to compensate for the frontal dysfunction, at least at an early stage of the disease, like in our patients. At this stage, even if some patients may complain of attentional difficulties, the impairment is quite subtle, as can be seen in the results of cognitive assessment (see S1 File and S1 Fig), or in Dujardin et al. [94]. This suggests that compensatory mechanisms may take place at these early stages of PD. This may explain the absence of significant modification of N2 amplitude and latency in our study, as well as the small behavioral differences between groups. Modifications of generators of the N2 favour this hypothesis. It can thus be suggested that the unusual N2 generators found in PD individuals at posterior sites could act as a compensating activity for a hypofunctioning of some frontal areas, leading to a relatively well-preserved execution of the task. Moreover, a compensatory effect of the dopaminergic treatment, as patients were on-drug at the time of the recording could also contribute to the absence of modification of the N2 latency and amplitude or the relative preservation of the behavioural features.

Nevertheless, this hypothesis does not explain why only the distracter-elicited P3 component is altered in PD–a finding that agrees well with the observed higher rate of false alarms to distracters in our patients, in the absence of any other behavioral abnormalities. The main difference between the stimuli studied here relates to the fact that before performing the task, the subjects received explicit instructions on (and thus expected) standard and target stimuli but not distracter stimuli. In fact, the subjects first became aware of the presence of distracter stimuli during the task itself. Inhibition of a response to an unexpected distracter required the subject to adapt his/her behavior. Impaired attention in PD patients has mainly been evidenced when a task requires internal control [3]. We suggest that in our present task, the distracter condition required more internal attentional control than the standard condition or even the target condition did. Resistance to interference by distracter stimuli may thus be impaired in PD and would contrast with relatively unaffected target detection and standard inhibition. However, this hypothesis needs to be confirmed by characterizing ERPs recorded during tasks that specifically compare internally and externally guided attentional control and context updating.

The present study had a number of limitations. Firstly, we investigated PD vs. HCs differences in the brain areas involved in early attentional process by analyzing the various N2 subcomponents. This objective prompted us to adopt a conventional, three-stimulus oddball paradigm that has been frequently used to study cognitive ERPs. We deliberately preferred this oddball paradigm to the other paradigms generally used to investigate inhibition (such as two-stimulus go/no-go tasks with a high target probability and no distracters [21]). Although go/no-go tasks have been frequently used to investigate the anterior N2 as an inhibition or error processing index, they prevent satisfactory investigation of early attentional processes for target or unexpected distracter detection. Nevertheless, our paradigm could have been improved by simultaneously manipulating the difficulty of target vs. standard discrimination and modifying the distracter, as shown in previous studies [95, 96]. Secondly, most of the patients had a mild form of PD with very mild cognitive disorders, as evidenced by the extensive cognitive assessment (see S1 File). Nevertheless, the PD patients' impairment in distracter processing was revealed by a higher commission rate in the oddball task. Even though recruitment of PD patients with more severe cognitive impairments may have better highlighted differences with respect to HCs, it would also have raised several potentially confounding issues. For example, a lack of specificity in the patients' cognitive disorders would interfere with the results. Later-stage PD would also have prevented good task performance and thus decreased the robustness of the ERP analysis. Thirdly, all the patients in the present study were assessed on-drug; this may represent a confounding factor, since dopamine replacement therapy could either minimize differences between PD patients and HCs in terms of performance and N2 features or modify the function of the corticosubcortical networks. Nevertheless, motor symptoms and a lack of motivation in off-drug patients would have jeopardized task performance and compromised our ERP analysis. Lastly, well-known, recurrent limitations of source localization studies include low spatial resolution (relative to fMRI, for example) and the error risk related to the use of statistical inferences. However, the use of high-resolution (128-channel) EEG markedly improved the spatial resolution and application of the swLORETA method reduces the localization error [15, 37]. Nevertheless, a multimodal, simultaneous EEG-fMRI study (combining swLORETA and an fMRI analysis) would constitute a more robust approach for identifying the neuro-anatomic substrates of attentional processes (as shown by Strobel et al.’s work [97]).

In conclusion, we investigated both common and specific sources of standard-, target- and distracter-elicited N2 subcomponents in PD patients and HCs during a three-stimulus visual oddball task. For all three types of stimuli, PD patients displayed fewer N2 generators in both the DLPFC and the ACC. The absence of significant differences between the generators for the three N2 subcomponents in patients suggested a lack of discrimination between stimuli during early-stage processing in PD. Our data suggest that regardless of the type of stimulus, early-stage attentional processes in the DLPFC and the ACC are impaired in PD. This impairment is probably related to the basal ganglia dysfunction responsible for altered cognitive control and mismatch detection, without a specific dysfunction of the implementation of selection. These abnormalities may underlie the impaired inhibition that is responsible for the attentional disorder seen in PD, as evidenced by our previous study of P3 components.

## Supporting Information

S1 Fig ERP waveforms at the N200 peak. ERP waveforms from Cz for standard stimuli (the thin grey line), target stimuli (the thick black line) and distracter stimuli (the thick grey line), with denotation N200 components (arrow). Representative data from a healthy control subject and a PD patient are shown on the left and the right, respectively.
(TIF)

S1 File Cognitive assessment description. (DOC)

S1 Table Results of both groups at the extensive cognitive assessment. Values are given as means and standard deviations. Group comparisons were performed using non-parametric Mann-Whitney tests. P values below 0.05 were considered to be statistically significant.
(DOC)

S2 Table Standard, distracter and target-elicited N2 amplitudes and latencies. Values are given as mean (standard deviation).
(DOC)

S3 Table Localization of the N2 generators in healthy controls and Parkinson's disease (PD) patients, on the basis of one sample t-tests. A. Standard-elicited N2, B. Distracter-elicited N2, C. Target-elicited N200. Talairach coordinates (Tx, Ty and Tz), anatomical location (gyrus and Brodmann area) and significance level. ACC: anterior cingulate cortex. PCC: posterior cingulate cortex.
(DOC)

S4 Table Localization of the specific N2 generators for the target stimuli and distracter stimuli, on the basis of paired t-tests in healthy controls (p<0.05). Talairach coordinates (T-x, T-y and T-z), anatomical location (gyrus and Brodmann area) and significance level. ACC: anterior cingulate cortex. PCC: posterior cingulate cortex.
(DOC)

S5 Table Localization of the specific N2 generators in healthy controls (on the left) and PD patients (on the right) for each stimulus, on the basis of two-sample t-tests (p<0.05). A. Standard-elicited N200, B. Distracter-elicited N200, C. Target-elicited N200. Talairach coordinates (T-x, T-y and T-z), anatomical location.
(DOC)
