# Supplementary figures and images for: Impaired Early Attentional Processes in Parkinson’s Disease: A High-Resolution Event-Related Potentials Study
Source: PLoS One. 2015 Jul 2;10(7):e0131654. doi: 10.1371/journal.pone.0131654 (PMC4489862; doi:10.1371/journal.pone.0131654)

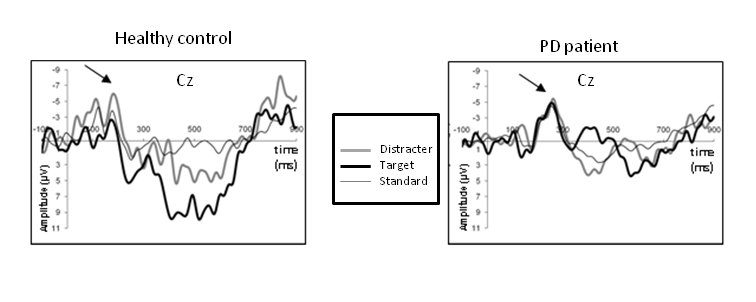

Supplement: S1 Fig — ERP waveforms from Cz for standard stimuli (the thin grey line), target stimuli (the thick black line) and distracter stimuli (the thick grey line), with denotation N200 components (arrow). Representative data from a healthy control subject and a PD patient are shown on the left and the right, respectively. (TIF) [file pone.0131654.s001.tif]
