# Supplementary material for: Impaired Early Attentional Processes in Parkinson’s Disease: A High-Resolution Event-Related Potentials Study
Source: PLoS One. 2015 Jul 2;10(7):e0131654. doi: 10.1371/journal.pone.0131654 (PMC4489862; doi:10.1371/journal.pone.0131654)
Supplement: S1 File — (DOC) [file pone.0131654.s002.doc]

**S1 File. Cognitive assessment description.**

**Method**

An extensive neuropsychological assessment was administered in order to encompass the cognitive domains that are sensitive to dysfunction in PD.

*Overall cognitive status* was assessed in terms of the score on the Mattis dementia rating scale [1].

*Memory* was assessed using (i) the forward and backward digit span and (ii) the French version of the Grober and Buschke 16-item free/cued word learning and recall test [2] . Performance was assessed in terms of the number of words (out of 16) immediately recalled at learning, the total number of words (out of 48) correctly recalled after the three free recall trials and the total number of words (out of 48) correctly recalled after the three free and cued recall trials.

*Attention and executive functions* were assessed using:

- An oral version of the Symbol Digit Modalities test [3]. Subjects were instructed to associate symbols with digits according to a key code. Performance was evaluated in terms of the number of correct responses given in 90 sec.

- A 50-item version of the Stroop word color test (to assess response inhibition). The procedure has been described fully elsewhere^4^. Performance was evaluated in terms of the time needed to complete the test's two phases (naming the color of dots and color names, respectively) and the number of errors in the interference phase (phase 2).

- A letter and number sequencing task, corresponding to an oral version of the Trail Making Test (to assess set shifting) [4]. Performance was evaluated in terms of the time needed to complete the test's two phases (baseline and alternation, respectively) and the number of errors in the alternation phase.

- A word generation task performed over 60 sec and in three conditions (to assess action initiation and working memory updating), i.e. phonemic (letter P), semantic (animals) and alternating (letter T and V, alternatively) conditions.

**Results**

S2 Table shows the mean (*SD*) results of both groups at the extensive neuropsychological assessment. Group comparisons revealed no significant difference, except that PD patients took significantly more time to complete the Symbol Digit Modalities test and the alternation phase of the letter and number sequencing task. (gyrus and Brodmann area) and significance level.

**References**

1. Mattis S., 1976. Mental status examination for organic mental syndrome in the elderly patient. In: Bellak L, Karasy T, eds. Geriatric Psychiatry. New York: Grune and Stratton, 77-121.

2. Van der Linden, M., Coyette, F., Poitrenaud, J., Kalafat, M., Calicis, C., Wyns, S., Adam, S., Agniel, A., Baisset-Mouly, C., Bardet, F., Desgranges, B., Deweer, B., Ergis, A.M., Gély-Nargeot, M.-C., Grymonprez, L., Juillerat, A.C., Mouly, C. Gely-Nargeot, M.C., Sellal. F. & Thomas, C., 2004. L'épreuve de rappel libre/rappel indicé à 16 items (RL/RI-16). In: Van der Linden M, GREMEM, eds. L'évaluation des troubles de la mémoire. Marseille: Solal,: 25-47.

3. Smith A., 1982. Symbol Digit Modalities Test (SDMT): Western Psychological Services.

4. Dujardin K., Defebvre L., Grunberg C., Becquet E., Destee A., 2001. Memory and executive function in sporadic and familial Parkinson's disease. Brain;124(Pt 2): 389-398.
