# Supplementary material for: Impaired Early Attentional Processes in Parkinson’s Disease: A High-Resolution Event-Related Potentials Study
Source: PLoS One. 2015 Jul 2;10(7):e0131654. doi: 10.1371/journal.pone.0131654 (PMC4489862; doi:10.1371/journal.pone.0131654)
Supplement: S1 Table — Values are given as means and standard deviations. Group comparisons were performed using non-parametric Mann-Whitney tests. P values below 0.05 were considered to be statistically significant. (DOC) [file pone.0131654.s003.doc]

| **Tasks** | **PD patients** | **Healthy Controls** | **p value (Mann-Whitney)** |
| --- | --- | --- | --- |
| *Mattis dementia rating scale (/144)* | 141.3 (2.7) | 142.1 (1.6) | 0.642 |
| *Forward Digit span* | 6.3 (1.3) | 6.3 (1) | 0.983 |
| *Backward Digit span* | 4.9 (1.8) | 4.1 (1.1) | 0.144 |
| *16-item free/cued word learning and recall test: immediate recall (/16)* | 15.5 0.9) | 15.9 (0.4) | 0.312 |
| *16-item free/cued word learning and recall test:Total Free Recall (/48)* | 32.1 (4.3) | 31.3 (3.1) | 0.63 |
| *16-item free/cued word learning and recall test:Total Free + Cued Recall (/48)* | 47.4 (0.9) | 46.8 (1.6) | 0.327 |
| *Symbol Digit Modalities test (number of correct responses)* | 46.7 (10.8) | 53.3 (4.8) | *0.027* |
| *Stroop word/color test: time to complete phase 1 (sec)* | 33.5 (4.2) | 32.2 (5.1) | 0.466 |
| *Stroop word/color test: time to complete phase 2 (sec)* | 54.7 (12.4) | 52 (9.8) | 0.455 |
| *Stroop word/color test: number of error in phase 2* | 1 (1.6) | 1.6 (1.5) | 0.187 |
| *Letter/Number sequencing: time to complete phase A (sec)* | 16.2 (11.9) | 9.3 (3.2) | 0.114 |
| *Letter/Number sequencing: time to complete phase B (sec)* | 34.2 (16.2) | 22.2 (4.9) | *0.024* |
| *Letter/Number sequencing: errors* | 0.3 (0.7) | 0.2 (0.4) | 0.858 |
| *Word generation task (60 sec): letter "P"* | 14.8 (5.2) | 16 (3.8) | 0.279 |
| *Word generation task (60 sec): Animals* | 21.1 (4.2) | 22.9 (6.2) | 0.204 |
| *Word generation task (60 sec): Alternating "T"/"V"* | 12.1 (4.2) | 13.9 (3.8) | 0.26 |

**S1 Table. Results of both groups at the extensive cognitive assessment**
