# Supplementary material for: Impaired Early Attentional Processes in Parkinson’s Disease: A High-Resolution Event-Related Potentials Study
Source: PLoS One. 2015 Jul 2;10(7):e0131654. doi: 10.1371/journal.pone.0131654 (PMC4489862; doi:10.1371/journal.pone.0131654)
Supplement: S2 Table — Values are given as mean (standard deviation). (DOC) [file pone.0131654.s004.doc]

**S2 Table. Standard, distracter and target-elicited N2 amplitudes and latencies**

|  |  | standard-elicited N2 | | |  | distracter-elicited N2 | | |  |  | target-elicited N2 | |
| --- | --- | --- | --- | --- | --- | --- | --- | --- | --- | --- | --- | --- |
|  |  | Fz | Cz | Pz |  | Fz | Cz | Pz |  | Fz | Cz | Pz |
| Amplitude | PD patients | -0.60 (2.37) | -0.62 (2.40) | 1.05 (1.85) |  | -0.22 (2.67) | 0.20 (2.74) | 0.86 (2.23) |  | 1.23 (2.98) | 1.14 (2.61) | -0.02 (1.68) |
| (µV) | Healthy controls | 0.06 (1.85) | -0.05 (1.7) | 0.26 (1.06) |  | 0.38 (2.83) | 0.06 (2.61) | 0.24 (1.58) |  | -0.17 (2.4) | -0.84 (1.68) | -0.66 (1.45) |
| Latency | PD patients | 251 (42) | 251 (45) | 258 (48) |  | 255 (50) | 257 (51) | 256 (55) |  | 255 (50) | 256 (51) | 253 (53) |
| (ms) | Healthy controls | 237 (57) | 237 (55) | 238 (57) |  | 230 (50) | 227 (51) | 228 (53) |  | 238 (64) | 238 (63) | 239 (65) |
