# Supplementary material for: Impaired Early Attentional Processes in Parkinson’s Disease: A High-Resolution Event-Related Potentials Study
Source: PLoS One. 2015 Jul 2;10(7):e0131654. doi: 10.1371/journal.pone.0131654 (PMC4489862; doi:10.1371/journal.pone.0131654)
Supplement: S3 Table — A. Standard-elicited N2, B. Distracter-elicited N2, C. Target-elicited N200. Talairach coordinates (Tx, Ty and Tz), anatomical location (gyrus and Brodmann area) and significance level. ACC: anterior cingulate cortex. PCC: posterior cingulate cortex. (DOC) [file pone.0131654.s005.doc]

**S3 Table. Localization of the N2 generators in healthy controls and Parkinson's disease (PD) patients, on the basis of one sample t-tests (p<0.001).**

| **Healthy controls** | | | | | | |  | **PD patients** | | | | | |
| --- | --- | --- | --- | --- | --- | --- | --- | --- | --- | --- | --- | --- | --- |
| **generators** | **Area (gyrus)** | **BA** |  | **Coordinates** |  | **T value** |  | **Area (gyrus)** | **BA** |  | **Coordinates** |  | **T value** |
|  |  |  | **Tx(mm)** | **Ty(mm)** | **Tz(mm)** |  |  |  |  | **Tx(mm)** | **Ty(mm)** | **Tz(mm)** |  |
| **A-Standard** | right PCC | *30* | 3 | -69 | 13 | 7.29689 |  | right caudate (head) |  | 10 | 6 | 3 | 6.56824 |
|  | right thalamus |  | 21 | -18 | 9 | 6.88434 |  | left middle occipital | *18* | -26 | -89 | 3 | 6.10915 |
|  | right insula | *13* | 41 | -27 | 17 | 6.73885 |  | left PCC | *30* | -17 | -59 | 14 | 5.943 |
|  | left caudate (head) |  | -10 | 16 | 4 | 6.33128 |  | right ACC | *24* | 9 | 36 | 8 | 5.5743 |
|  | right putamen |  | 31 | -16 | -8 | 5.71357 |  | right occipital cuneus | *19* | 23 | -90 | 29 | 5.49026 |
|  | right parietal angularis | *39* | 42 | -59 | 32 | 5.62818 |  | left putamen |  | -29 | -11 | 1 | 5.38182 |
|  | left inferior temporal | *20* | -39 | -17 | -15 | 5.17937 |  | right occipital lingual | *18* | 13 | -66 | -4 | 5.35407 |
|  | left frontal precentral | *6* | -39 | -7 | 38 | 5.0688 |  | right superior occipital | *19* | 32 | -69 | 22 | 5.20077 |
|  | left occipital fusiformis | *19* | -28 | -57 | -11 | 4.85466 |  | right frontal precentral | *6* | 40 | -6 | 46 | 5.04432 |
|  | right middle temporal | *21* | 61 | -33 | -9 | 4.79408 |  | right insula | *13* | 41 | -27 | 17 | 4.99429 |
|  | right inferior parietal | *40* | 41 | -35 | 34 | 4.68744 |  | right ACC | *32* | 10 | 23 | 31 | 4.97367 |
|  | right medial frontal | *10* | 18 | 57 | -1 | 4.66634 |  | right parietal postcentral | *2* | 41 | -29 | 35 | 4.87743 |
|  | left superior parietal | *7* | -17 | -54 | 60 | 4.44382 |  | right middle occipital | *18* | 33 | -87 | 3 | 4.85302 |
|  | right ACC | *24* | 1 | -5 | 29 | 4.19126 |  | left superior temporal | *22* | -58 | -51 | 15 | 4.80668 |
|  | right inferior frontal | *9* | 40 | 4 | 29 | 4.15728 |  | left inferior occipital | *18* | -27 | -87 | -13 | 4.78343 |
|  | left middle temporal | *21* | -58 | -60 | 6 | 3.99489 |  | left central precuneus | *7* | -7 | -73 | 49 | 4.75693 |
|  | right frontal precentral | *6* | 40 | -6 | 55 | 3.89771 |  | right inferior parietal | *40* | 38 | -52 | 33 | 4.59469 |
|  | left superior frontal | *6* | -19 | -9 | 64 | 3.87948 |  | right middle temporal | *21* | 61 | -34 | -2 | 4.49987 |
|  | right frontal precentral | *4* | 21 | -23 | 62 | 3.87117 |  | left inferior parietal | *40* | -48 | -44 | 52 | 4.36843 |
|  | left frontal precentral | *4* | -38 | -25 | 63 | 3.43251 |  | right inferior frontal | *9* | 40 | 4 | 29 | 4.31857 |
|  | right paracentral | *6* | 2 | -33 | 62 | 3.43016 |  | right inferior temporal | *19* | 52 | -65 | -4 | 4.28711 |
|  |  |  |  |  |  |  |  | right superior frontal | *9* | 19 | 43 | 33 | 4.2136 |
|  |  |  |  |  |  |  |  | left inferior temporal | *20* | -59 | -26 | -16 | 4.19649 |
|  |  |  |  |  |  |  |  | left frontal precentral | *4* | -59 | -7 | 29 | 3.83929 |
|  |  |  |  |  |  |  |  | left inferior frontal | *45* | -51 | 34 | 6 | 3.62491 |
|  |  |  |  |  |  |  |  | left middle frontal | *6* | -29 | -16 | 55 | 3.44684 |
|  |  |  |  |  |  |  |  |  |  |  |  |  |  |
| **B-Distracter** | right PCC | *31* | 3 | -60 | 23 | 5.22356 |  | right caudate |  | 10 | 5 | 12 | 4.58648 |
|  | left parahippocampal | *36* | -39 | -26 | -16 | 5.15335 |  | right superior occipital | *19* | 32 | -69 | 22 | 4.58372 |
|  | left putamen |  | -30 | -3 | -6 | 5.08811 |  | right PCC | *29* | 2 | -48 | 6 | 4.49744 |
|  | left ACC | *32* | -1 | 45 | 6 | 5.00059 |  | left thalamus |  | -19 | -19 | 9 | 4.37163 |
|  | right thalamus |  | 21 | -18 | 9 | 4.98931 |  | left caudate (body) |  | -10 | 5 | 12 | 4.31701 |
|  | right parahippocampal | *36* | 31 | -34 | -9 | 4.71699 |  | right thalamus |  | 11 | -27 | 8 | 4.27698 |
|  | right caudate (body) |  | 10 | 5 | 12 | 4.67386 |  | right anterior precuneus | *7* | 3 | -63 | 50 | 4.11876 |
|  | right insula | *13* | 41 | -18 | 9 | 4.57305 |  | left ACC | *32* | -1 | 23 | 31 | 4.02816 |
|  | right inferior temporal | *20* | 40 | -14 | -31 | 4.54748 |  | right inferior occipital | *18* | 43 | -86 | -5 | 3.91953 |
|  | left central precuneus | *19* | -16 | -83 | 39 | 4.46841 |  | right insula | *13* | 30 | 6 | 12 | 3.87275 |
|  | right frontal precentral | *4* | 21 | -23 | 62 | 4.29895 |  | left middle occipital | *18* | -26 | -89 | 3 | 3.64718 |
|  | right temporal angulais | *39* | 52 | -69 | 31 | 4.11667 |  | right middle frontal | *8* | 39 | 23 | 40 | 3.57022 |
|  | right paracentral | *6* | 2 | -33 | 62 | 4.09998 |  | right middle temporal | *21* | 70 | -24 | -1 | 3.55159 |
|  | left frontal precentral | *4* | -39 | -15 | 46 | 4.07624 |  | right frontal precentral | *6* | 40 | -6 | 46 | 3.48665 |
|  | left occipital fusiformis | *19* | -37 | -67 | -11 | 4.05681 |  |  |  |  |  |  |  |
|  | left middle temporal | *39* | -37 | -70 | 14 | 4.00649 |  |  |  |  |  |  |  |
|  | right superior temporal | *22* | 61 | -47 | 15 | 3.94269 |  |  |  |  |  |  |  |
|  | left superior frontal | *6* | -19 | -9 | 64 | 3.8069 |  |  |  |  |  |  |  |
|  | right occipital cuneus | *18* | 23 | -97 | 2 | 3.63547 |  |  |  |  |  |  |  |
|  | left middle temporal | *21* | -58 | -60 | 6 | 3.53964 |  |  |  |  |  |  |  |
|  |  |  |  |  |  |  |  |  |  |  |  |  |  |
| **C-Target** | right supramarginalis | *40* | 41 | -39 | 34 | 5.18401 |  | right putamen |  | 20 | 17 | 4 | 4.72149 |
|  | left occipital lingual | *18* | -27 | -67 | -12 | 4.63538 |  | left caudate (head) |  | -10 | 16 | 4 | 4.35664 |
|  | left uncus | *28* | -10 | -1 | -30 | 4.489 |  | left occipital lingual | *18* | -7 | -58 | 5 | 4.35304 |
|  | left medial frontal | *25* | -10 | 8 | -13 | 4.48144 |  | right thalamus |  | 1 | -10 | 1 | 4.14127 |
|  | left middle occipital | *18* | -26 | -89 | 3 | 4.37553 |  | right ACC | *32* | 9 | 35 | 14 | 4.02099 |
|  | left occipital fusiformis | *37* | -28 | -47 | -10 | 4.36766 |  | right frontal precentral | *6* | 40 | -6 | 46 | 3.85726 |
|  | right superior temporal | *42* | 61 | -26 | 17 | 4.33542 |  | right occipital lingual | *18* | 33 | -75 | -13 | 3.84591 |
|  | right ACC | *32* | 9 | 34 | 23 | 4.30799 |  | right superior temporal | *42* | 61 | -26 | 17 | 3.81295 |
|  | left anterior precuneus | *7* | -17 | -74 | 34 | 4.23674 |  | right parietal postcentral | *2* | 41 | -29 | 35 | 3.81027 |
|  | right superior occipital | *19* | 32 | -69 | 22 | 4.21352 |  | left middle occipital | *18* | -26 | -89 | 3 | 3.77064 |
|  | left anterior precuneus | *7* | -7 | -52 | 42 | 4.14409 |  | right middle frontal | *9* | 49 | 24 | 31 | 3.72865 |
|  | right frontal precentral | *4* | 60 | -12 | 36 | 4.13942 |  | right frontal precentral | *6* | 59 | 5 | 29 | 3.61503 |
|  | right thalamus |  | 21 | -18 | 9 | 4.12768 |  | left inferior parietal | *40* | -38 | -52 | 33 | 3.50658 |
|  | right superior frontal | *10* | 29 | 45 | 15 | 3.91121 |  | left superior temporal | *39* | -48 | -49 | 7 | 3.48095 |
|  | right middle temporal | *21* | 60 | -23 | -9 | 3.85811 |  |  |  |  |  |  |  |
|  | left frontal precentral | *6* | -39 | -8 | 47 | 3.812 |  |  |  |  |  |  |  |
|  | left middle temporal | *22* | -48 | -38 | -1 | 3.80804 |  |  |  |  |  |  |  |
|  | right superior frontal | *8* | 9 | 42 | 42 | 3.77555 |  |  |  |  |  |  |  |
|  | left inferior frontal | *9* | -50 | 11 | 30 | 3.75001 |  |  |  |  |  |  |  |
|  | right middle frontal | *6* | 30 | -6 | 46 | 3.72033 |  |  |  |  |  |  |  |
|  | right uncus | *28* | 20 | 1 | -30 | 3.71404 |  |  |  |  |  |  |  |
|  | left frontal precentral | *4* | -38 | -25 | 63 | 3.67741 |  |  |  |  |  |  |  |
|  | right superior frontal | *6* | 1 | 20 | 58 | 3.5745 |  |  |  |  |  |  |  |
|  | left superior frontal | *6* | -19 | -1 | 65 | 3.4788 |  |  |  |  |  |  |  |
