# Supplementary material for: Impaired Early Attentional Processes in Parkinson’s Disease: A High-Resolution Event-Related Potentials Study
Source: PLoS One. 2015 Jul 2;10(7):e0131654. doi: 10.1371/journal.pone.0131654 (PMC4489862; doi:10.1371/journal.pone.0131654)
Supplement: S4 Table — Talairach coordinates (T-x, T-y and T-z), anatomical location (gyrus and Brodmann area) and significance level. ACC: anterior cingulate cortex. PCC: posterior cingulate cortex. (DOC) [file pone.0131654.s006.doc]

**S4 Table. Localization of the specific N2 generators for the target stimuli and distracter stimuli, on the basis of paired t-tests in healthy controls (p<0.05).**

| generators | Area (gyrus) | BA |  | Coordinates |  | T-score |
| --- | --- | --- | --- | --- | --- | --- |
|  |  |  | T-x(mm) | T-y(mm) | T-z(mm) |  |
| Distracter-standard | left PCC | *30* | 23 | -68 | 13 | 2.78073 |
|  | left occipital lingual | *18* | -17 | -68 | 5 | 2.61503 |
|  | left parahippocampus | *28* | -21 | -15 | -23 | 2.52666 |
|  | left uncus | *20* | -29 | -16 | -31 | 2.49995 |
|  | right parahippocampus | *36* | 31 | -34 | -9 | 2.44518 |
|  | right occipital lingual | *18* | 23 | -67 | 4 | 2.41992 |
|  | right medial frontal | *25* | 20 | 7 | -13 | 2.36107 |
|  | right medial frontal | *10* | 18 | 56 | 7 | 2.35151 |
|  | right thalamus |  | 21 | -18 | 9 | 2.26016 |
|  | right cuneus | *18* | 14 | -99 | 11 | 2.25743 |
|  | right superior temporal | *21* | 51 | -25 | -1 | 2.21587 |
|  | left precentral | *4* | -47 | -15 | 46 | 2.14597 |
|  | right fusiformis | *20* | 60 | -7 | -23 | 2.01333 |
|  | left inferior parietal |  | -58 | -32 | 35 | 1.99544 |
|  | right superior temporal | *22* | 59 | 9 | -6 | 1.97806 |
|  | right angularis | *39* | 52 | -69 | 31 | 1.92658 |
|  | left superior frontal | *6* | -33 | 13 | 48 | 1.88629 |
|  | right supramarginalis | *40* | 51 | -39 | 34 | 1.76026 |
|  | left middle occipital | *18* | -26 | -89 | 3 | 1.70229 |
| Target-standard | right angularis | *39* | 52 | -69 | 31 | 2.34539 |
|  | right superior frontal | *10* | 28 | 55 | 16 | 2.25457 |
|  | right middle occipital | *19* | 33 | -89 | 20 | 2.11909 |
|  | left rectal frontal | *11* | -10 | 19 | -20 | 2.10773 |
|  | right ACC | *32* | 9 | 35 | 14 | 2.07624 |
|  | left frontal precentral | *6* | -59 | -7 | 38 | 2.07564 |
|  | left middle frontal | *6* | -40 | 1 | 39 | 2.02218 |
|  | left central precuneus | *19* | -16 | -83 | 39 | 2.01237 |
|  | right superior temporal | *21* | 51 | -25 | -1 | 1.92916 |
|  | left superior parietal | *7* | -17 | -64 | 59 | 1.91185 |
|  | left supramarginalis | *40* | -56 | -37 | 35 | 1.88053 |
|  | right fusiformis | *37* | 51 | -43 | -18 | 1.86611 |
|  | left uncus | *28* | -10 | -1 | -30 | 1.84511 |
|  | right uncus | *36* | 20 | -7 | -31 | 1.80501 |
