# Supplementary material for: Impaired Early Attentional Processes in Parkinson’s Disease: A High-Resolution Event-Related Potentials Study
Source: PLoS One. 2015 Jul 2;10(7):e0131654. doi: 10.1371/journal.pone.0131654 (PMC4489862; doi:10.1371/journal.pone.0131654)
Supplement: S5 Table — A. Standard-elicited N200, B. Distracter-elicited N200, C. Target-elicited N200. Talairach coordinates (T-x, T-y and T-z), anatomical location. (DOC) [file pone.0131654.s007.doc]

**S5 Table. Localization of the specific N2 generators in healthy controls (on the left) and PD patients (on the right) for each stimulus, on the basis of two-sample t-tests (p<0.05).**

| Healthy controls | | | | | | |  | PD patients | | | | | |
| --- | --- | --- | --- | --- | --- | --- | --- | --- | --- | --- | --- | --- | --- |
| generators | Area (gyrus) | BA | Coordinates | | | T-score |  | Area (gyrus) | BA | Coordinates | | | T-score |
|  |  |  | Tx(mm) | Ty(mm) | Tz(mm) |  |  |  |  | Tx(mm) | Ty(mm) | Tz(mm) |  |
| A-standard | right inferior parietal | *40* | 51 | -27 | 22 | 3.2568 |  | right middle occipital | *19* | 33 | -88 | 12 | 3.2341 |
|  | right frontal precentral | *6* | 50 | -5 | 37 | 2.66583 |  | left occipital cuneus | *19* | -6 | -91 | 30 | 2.65073 |
|  | left medial frontal | *10* | -2 | 65 | 8 | 2.29918 |  | left inferior parietal | *40* | -48 | -32 | 35 | 1.93826 |
|  | left ACC | *32* | -11 | 33 | 23 | 2.13003 |  | left parietal postcentral | *1* | -48 | -24 | 54 | 1.87515 |
|  | right inferior frontal | *44* | 49 | 17 | 12 | 1.92791 |  | left occipital lingual | *18* | -17 | -66 | -12 | 1.85264 |
|  | left insula | *13* | -30 | 14 | 13 | 1.84285 |  | left temporal fusiformis | *37* | -38 | -56 | -19 | 1.79436 |
|  | right inferior frontal | *47* | 39 | 19 | -13 | 1.8367 |  | right central precuneus | *19* | 42 | -71 | 40 | 1.77282 |
| B-distracter | right superior temporal | *42* | 61 | -25 | 8 | 2.57582 |  | right superior occipital | *19* | 42 | -75 | 28 | 3.27423 |
|  | right inferior frontal | *47* | 39 | 19 | -13 | 2.10951 |  | right superior temporal | *39* | 62 | -58 | 23 | 2.52969 |
|  | left superior frontal | *10* | -32 | 55 | -1 | 1.99084 |  | right middle occipital | *19* | 33 | -88 | 12 | 1.98834 |
|  | right ACC | *32* | 8 | 46 | 6 | 1.74257 |  | left anterior precuneus | *7* | -28 | -46 | 52 | 1.80776 |
|  |  |  |  |  |  |  |  | right anterior precuneus | *7* | 10 | -63 | 59 | 1.76559 |
| C- target | left superior temporal | *22* | -60 | 4 | 3 | 2.28588 |  | right occipital lingual | *18* | 3 | -67 | -4 | 3.55458 |
|  | left medial frontal | *10* | -2 | 65 | 8 | 2.15781 |  | left frontal precentral | *4* | -29 | -15 | 46 | 2.32879 |
|  | left inferior frontal | *45* | -30 | 25 | 5 | 1.97496 |  | left middle occipital | *18* | -26 | -90 | 12 | 2.00216 |
|  | right frontal precentral | *4* | 50 | -12 | 36 | 1.88305 |  | left parietal postcentral | *3* | -28 | -33 | 53 | 1.79214 |
|  | right superior temporal | *22* | 60 | -16 | -1 | 1.80277 |  |  |  |  |  |  |  |
